# Estimating Trends in Cardiovascular Disease Risk for the EXPOSE (Explaining Population Trends in Cardiovascular Risk: A Comparative Analysis of Health Transitions in South Africa and England) Study: Repeated Cross-Sectional Study

**DOI:** 10.2196/64893

**Published:** 2025-01-20

**Authors:** Shaun Scholes, Jennifer S Mindell, Mari Toomse-Smith, Annibale Cois, Kafui Adjaye-Gbewonyo

**Affiliations:** 1 Department of Epidemiology and Public Health University College London London United Kingdom; 2 National Centre for Social Research London United Kingdom; 3 Burden of Disease Research Unit South African Medical Research Council Cape Town South Africa; 4 Division of Epidemiology and Biostatistics School of Public Health University of Cape Town Cape Town South Africa; 5 Faculty of Education Health and Human Sciences University of Greenwich London United Kingdom

**Keywords:** data harmonization, cardiovascular disease, CVD, CVD risk scores, trends, cross-country comparisons, public health, England, South Africa

## Abstract

**Background:**

Cardiovascular diseases (CVDs) are the leading cause of death globally. Demographic, behavioral, socioeconomic, health care, and psychosocial variables considered risk factors for CVD are routinely measured in population health surveys, providing opportunities to examine health transitions. Studying the drivers of health transitions in countries where multiple burdens of disease persist (eg, South Africa), compared with countries regarded as models of “epidemiologic transition” (eg, England), can provide knowledge on where best to intervene and direct resources to reduce the disease burden.

**Objective:**

The EXPOSE (Explaining Population Trends in Cardiovascular Risk: A Comparative Analysis of Health Transitions in South Africa and England) study analyzes microlevel data collected from multiple nationally representative population health surveys conducted in these 2 countries between 1998 and 2017. Creating a harmonized dataset by pooling repeated cross-sectional surveys to model trends in CVD risk is challenging due to changes in aspects such as survey content, question wording, inclusion of boost samples, weighting, measuring equipment, and guidelines for data protection. This study aimed to create a harmonized dataset based on the annual Health Surveys for England to estimate trends in mean predicted 10-year CVD risk (primary outcome) and its individual risk components (secondary outcome).

**Methods:**

We compiled a harmonized dataset to estimate trends between 1998 and 2017 in the English adult population, including the primary and secondary outcomes, and potential drivers of those trends. Laboratory- and non–laboratory-based World Health Organization (WHO) and Globorisk algorithms were used to calculate the predicted 10-year total (fatal and nonfatal) CVD risk. Sex-specific estimates of the mean 10-year CVD risk and its components by survey year were calculated, accounting for the complex survey design.

**Results:**

Laboratory- and non–laboratory-based 10-year CVD risk scores were calculated for 33,628 and 61,629 participants aged 40 to 74 years, respectively. The absolute predicted 10-year risk of CVD declined significantly on average over the last 2 decades in both sexes (for linear trend; all *P*<.001). In men, the mean of the laboratory-based WHO risk score was 10.1% (SE 0.2%) and 8.4% (SE 0.2%) in 1998 and 2017, respectively; corresponding figures in women were 5.6% (SE 0.1%) and 4.5% (SE 0.1%). In men, the mean of the non–laboratory-based WHO risk score was 9.6% (SE 0.1%) and 8.9% (SE 0.2%) in 1998 and 2017, respectively; corresponding figures in women were 5.8% (SE 0.1%) and 4.8% (SE 0.1%). Predicted CVD risk using the Globorisk algorithms was lower on average in absolute terms, but the pattern of change was very similar. Trends in the individual risk components showed a complex pattern.

**Conclusions:**

Harmonized data from repeated cross-sectional health surveys can be used to quantify the drivers of recent changes in CVD risk at the population level.

## Introduction

The global burden of noncommunicable diseases is increasing [1,2]. Cardiovascular diseases (CVDs) in particular lead globally in terms of causes of mortality [3] and often share characteristics with other major noncommunicable diseases. For instance, they tend to increase with age and can be influenced by healthy lifestyle behaviors as well as other demographic, social, and environmental factors. Along with questions on the presence, diagnosis, and treatment of chronic disease–related conditions, population health surveys conducted at regular intervals often include measures of risk factors for CVD, thus providing opportunities to study health transitions.

Understanding the drivers of epidemiological transition in countries that have not followed predicted paths (eg, South Africa) compared with those that have served as examples (eg, England) can provide knowledge on where best to intervene and direct resources to reduce disease burden. The EXPOSE (Explaining Population Trends in Cardiovascular Risk: A Comparative Analysis of Health Transitions in South Africa and England) study uses participant-level data from nationally representative health surveys to examine health transitions by identifying and quantifying the drivers of trends in CVD risk in a middle-income country such as South Africa compared with a high-income nation such as England. Complete details about the EXPOSE study are available in the study protocol [4] and on the study website [5].

To enable empirical investigation of temporal trends in CVD risk, the first phase of the EXPOSE study was to compile harmonized datasets from the national health surveys conducted in South Africa and England [4]. Since 1991, the Health Survey for England (HSE) has monitored the health of the public in England, including regular updates on trends in key indicators such as smoking, physical activity (PA), overweight and obesity, hypertension, diabetes, and self-reported physician-diagnosed CVD [6]. Creating a harmonized dataset from the annual HSE surveys conducted over 2 decades (1998-2017) to model changes in CVD risk over time and decompose its variation (the later phases of the EXPOSE study) was a daunting task due to changes over time in aspects such as survey content, sampling design (inclusion of boost samples for population subgroups), question wording (eg, through changes in public health policy recommendations), introduction of nonresponse weighting, changes in measuring equipment (eg, changes in blood pressure [BP] monitors), and more stringent data release guidelines for protecting participant anonymity.

Herein, we describe the methods and procedures used to painstakingly compile the harmonized dataset for England, enabling the modeling of trends in CVD risk in adults and the investigation of the factors driving the trends. We anticipate that the dataset will be a valuable resource for the wider research community in the United Kingdom and worldwide (eg, by avoiding duplication of effort). The code for harmonizing and appending the England surveys for others to use in future research is publicly available through the study website [5] and from DataFirst [7]. For the presentation of early results, we provide sex-specific estimates of the mean total (fatal and nonfatal) 10-year CVD risk and its individual risk components (eg, BP, smoking, and physician-diagnosed diabetes) by survey year over 2 decades (1998-2017), accounting for the complex survey design.

## Methods

### The HSE

Data for England were drawn from the HSE, conducted from 1998 to 2017. The HSE is an annual cross-sectional, general population survey of individuals living in private households, with a new sample of addresses selected each year using random multistage stratified probability sampling. Complete details about the HSE, including its origins, sampling design, study content, and data availability, are provided in the “Cohort Profile: The Health Survey for England” [6].

Data collection for each survey was conducted continuously throughout the year, starting in January, to minimize seasonal effects. The process was carried out in 2 stages. The first stage was a computer-assisted health interview, including questions about sociodemographic factors, diagnosed health conditions, self-rated general health and illness, health-related lifestyle behaviors, and direct measurements of height and weight, by trained interviewers. The second stage was a nurse visit, including questions regarding current use of prescribed medicines, BP and other anthropometric measurements (eg, waist and hip circumference), and collection of nonfasting blood samples (eg, glycated hemoglobin [HbA_1c_] and cholesterol). Only those participants who completed the interview were eligible for the nurse visit. Interviews and nurse visits took place in the participants’ home. All adults (maximum 10) in selected households were eligible to take part; the percentage of eligible households participating ranged from 74% in 1998 to 59% in 2016.

The survey usually focuses on multiple health issues. The inclusion of a set of “core” questions and measurements each year (or repeated at regular intervals) provides consistency that is important for studying temporal trends in key health indicators. Some surveys included a greater focus on different single health topics, including PA and fitness in 2008 [8] and respiratory health in 2010 [9]. In a number of years, sampling was boosted to study specific subgroups of the population, including ethnic minority groups in 1999 [10] and 2004 [11], persons living in care homes in 2000 [12], children and young adults in 2002 [13], and persons aged ≥65 years living in private households in 2005 [14]. During these years, a smaller sample of the general population was also selected, with reduced survey content typically limited to the core set of questions and measurements (height and weight).

Through the combination of a health interview and health examination, data from the HSE can be used to investigate both diagnosed and undiagnosed disease at a point in time; a key strength therefore is that each sample is not selected based on health care use [15].

### Ethical Considerations

Each selected address for the HSE receives an advance letter introducing the survey and informing recipients that an interviewer will be visiting to request permission for an interview. Individual interviews are conducted with adults who give verbal informed consent. At the end of individual interviews, participants are asked for agreement to a follow-up visit by a trained nurse. Written consent is obtained for collection of nonfasting blood samples. The advance letters and information leaflets clearly state that participation in the survey is voluntary. Participants are also informed that they may choose not to answer specific questions, withdraw or stop at any time, or refuse any particular measurement if they wish. Interviewers and nurses will often repeat this information in their introductions, when they are setting up appointments, and throughout the interview as necessary. In fact, many individuals choose not to participate in the survey. Others may refuse to answer specific questions, discontinue the interview midway, or decline physical measurements. It is also standard practice to conduct interviews and nurse visits sometime after an appointment has been made so that individuals have a chance to reflect on their agreement before the appointment takes place. The procedures used in the HSE to obtain informed consent are very closely scrutinized by a National Health Service ethics committee each year (complete details are available in the annual HSE “Methods and documentation” reports). Information leaflets and both the content and wording of questionnaires are also carefully reviewed by the ethics committees.

The original data collection was approved each year by a National Health Service research ethics committee. The present analysis did not receive approval from a research ethics committee. The secondary analysis did not need ethical approval, as we used publicly available datasets [16-33]. The authors had permission to use the data.

### Creating a Harmonized Dataset

#### Selection of Participants for Inclusion

In the survey years including minority ethnic boost samples (1999 and 2004), nurse visits were offered to participants in the target minority ethnic groups only. As systolic BP (SBP, a component of cardiovascular risk scores) was measured during the nurse visit, the harmonized dataset does not include data from the 1999 and 2004 surveys. In addition, we excluded data from 2000 as the question on diagnosed diabetes was not included (also a component of CVD risk), and we included only those participants selected as part of the general population sample in the boost year of 2002. Taken together, the datasets covered 17 cross-sections of the adult population spanning the 20-year period from 1998 to 2017: these datasets are available to registered users via the UK Data Service and were compiled and appended to create the harmonized dataset [16-33].

#### CVD Risk Algorithms

##### Overview

###### Background

The predicted 10-year cardiovascular risk for HSE participants was calculated using laboratory-based and non–laboratory-based algorithms. Risk algorithms such as the Framingham Risk Score and those developed in England and Wales using the QResearch database are widely used in clinical and other settings to predict the risk of a future CVD event based on a number of laboratory results (eg, blood samples) and other demographic and self-reported risk factors [34]. Non–laboratory-based algorithms, based on physical examination and self-reported data, were developed for use in low-resource environments where laboratory-based measures may be difficult to obtain. In this study, we selected the World Health Organization (WHO) [35] and Globorisk [36,37] CVD risk algorithms for several reasons. Both are “global” models, accounting for differences in levels of CVD risk factors and event rates across populations, making them applicable to low-, middle-, and high-income countries. Both algorithms include the “traditional” CVD risk factors—age, sex, SBP, current smoking, diabetes, total cholesterol, and BMI—that are available in both the HSE and in South African datasets such as the Demographic and Health Surveys and the South Africa National Health and Nutrition Examination Survey, thereby fitting in line with the objective of comparing health transitions (using CVD risk as a case study) in these 2 countries. Finally, the statistical code for both algorithms is openly accessible to calculate the predicted 10-year CVD risk for participants in health surveys such as the HSE.

Both algorithms calculate the predicted 10-year risk of CVD, expressed as a proportion or a percentage, based on (1) an individual’s risk factor profile (eg, age, current smoking status, BP, total cholesterol, and diabetes history) and (2) the average CVD risk in the target population based on population levels of risk factors (obtained from national health surveys) and rates of CVD. Model derivation and recalibration were performed in both approaches in a broadly similar fashion. At the model derivation stage, individual-level data from prospective cohort studies were used to estimate hazard ratios (HRs) for each risk factor; these quantify the proportional effect of risk factors on CVD risk over the follow-up period. At the model recalibration stage, average risk factor levels and annual CVD event rates were reset to the levels observed in the target population to bring predicted risks in line with observed risks [37].

###### WHO Risk Score

The WHO algorithm predicts 10-year risk for the combined outcome of fatal and nonfatal CVD based on the revised WHO CVD risk models that have been recalibrated to reflect the expected 10-year risk in contemporary populations in 21 Global Burden of Disease (GBD) regions [35].

Risk prediction models were derived using individual participant data (aged 40-80 years with no baseline CVD) from 85 prospective cohorts mostly from high-income countries in the Emerging Risk Factors Collaboration. Follow-up was until the first CVD event; outcomes were censored if cases were lost to follow-up, died from non-CVD causes, or reached 10 years of follow-up. Variables were considered for inclusion in the risk models if they were known to predict CVD in diverse populations, were available in recent national health surveys for model recalibration within GBD regions, and could be measured at a low cost in low- and middle-income countries.

A laboratory-based CVD model included age, current smoking status, SBP, diabetes history, and total cholesterol; a non–laboratory-based model replaced diabetes and total cholesterol with BMI. Sex-specific models were fitted separately for (1) coronary heart disease (CHD; fatal-plus-nonfatal myocardial infarction or CHD death) and (2) fatal-plus-nonfatal stroke outcomes to enable separate recalibration before combination in a single equation for CVD [35]. HRs were estimated using Cox proportional hazards models, stratified by study and with duration (time-in-study) as the time scale. Interaction terms allowed the proportional effects of risk factors on the risk of CVD to vary by age (as evidence suggests that their impact declines with age).

Models were then recalibrated to the contemporary circumstances of the 21 GBD regions. The recalibration process is broadly similar for the WHO and Globorisk algorithms and involves resetting the average levels of risk factors and CVD risk to the levels observed in the target population. The input data and the steps involved in the model recalibration process, drawing largely on the worked example by the Cohorts Consortium of Latin America and the Caribbean [38], are described as follows.

Input data for model recalibration comprises (1) an individual’s risk factor profile (eg, age, sex, SBP, and current smoking status); (2) region-, sex-, and age-specific mean risk factor levels (eg, mean SBP and prevalence of current smoking); and (3) region-, sex-, and age-specific annual rates of CVD events. For the WHO algorithm, region-specific risk factor values were estimated by averaging country-specific levels provided by the Noncommunicable Disease Risk Factor Collaboration [39-43]; CVD incidence rates were obtained from the 2017 update of the GBD study [44,45].

The following steps in the model recalibration process refer to calculations performed separately for each year of follow-up over a period of 10 years (year 0 to year 9). First, for each risk factor, the difference (“distance”) is calculated between an individual’s risk factor profile and the group-specific mean risk factor levels. Second, for each risk factor, the distance is multiplied by the main coefficient (log HR) of the corresponding risk factor from the relevant (outcome-specific) Cox regression model. Third, for the risk factors whose proportional effect on the outcome varies by age, the distance (eg, individual SBP minus population mean SBP) is multiplied by the coefficient (log HR) of the interaction term and by the individual’s age (eg, for someone aged 60 years at year 0 through to age 69 years at year 9). Fourth, for each risk factor, the products obtained from steps 2 and 3 are summed and then exponentiated to calculate the risk factor–specific HR. Fifth, the risk-factor specific HRs are multiplied to compute the joint HR. Sixth, the 1-year risk of CVD is calculated as the product of the joint HR and the group-specific annual CVD event rate. Seventh, the 1-year survival is calculated as the exponential of the negative value of the 1-year risk of CVD (eg, a 1-year CVD risk of 0.06 translates to a 1-year survival of exp(–0.06)=0.942).

In the eighth stage, the cumulative survival is calculated as the product of the 1-year survival in year T and the survival in year T–1. In the ninth and final stage, the cumulative CVD risk is calculated as 1 minus the cumulative survival.

The cumulative CVD risk in the final year of follow-up (year 9) is the predicted *absolute* 10-year CVD risk. For example, based on a survey participants’ risk factor profile, a CVD risk of 9% can be interpreted as slightly less than a 1 in 10 chance of having a CVD event in the next 10 years. To facilitate interpretation, CVD risk scores are often categorized into groups such as “very low” (<5%), “low” (5%-10%), “moderate” (10%-20%), “high” (20%-30%), and “very high” (≥30%), and these cutoffs are often used in applications to estimate the proportion of individuals at high absolute CVD risk.

The individual risk factor components of the WHO CVD risk scores and the HSE survey years available for the calculation of CVD risk scores are summarized in Textbox 1. Laboratory-based WHO CVD risk scores are calculated using complete risk factor profile data on sex, age, current smoking status, SBP, history of diabetes, and total cholesterol. (To be comparable with South African data, diabetes status in this study was defined using only self-reported physician-diagnosed diabetes). The non–laboratory-based risk score replaces diabetes and total cholesterol with BMI.

Calculation of CVD risk in our study was limited to participants aged 40 to 74 years. Data on all components of the laboratory-based risk score were available in 1998, 2003, 2006, and from 2009 onward; all components of the non–laboratory-based score were available in 1998, 2001 to 2003, and from 2005 onward. In 2006, participants aged ≥65 years were allocated at random to either (1) the CVD (including diabetes) and short PA modules or (2) the long PA module but not the CVD module. Adults aged 16-64 years completed both the CVD and long PA modules. Herein, for the presentation of CVD trends, components were set to missing for a small number of participants with the following outlying values: *SBP* (<60 mm Hg or >270 mm Hg), *height* (<1.2 m or >2.2 m), *weight* (men: <35 kg or >250 kg; women: <25 kg or >250 kg), *BMI* (<10 kg/m^2^), and *total cholesterol* (<1.8 mmol/L or >20 mmol/L).

Total (ie, fatal and nonfatal) CVD risk scores for participants with valid data on all the relevant components (ie, complete cases) were calculated using the Stata (version 18.0; StataCorp) program *whocvdrisk*. A 10-year risk time was specified, with Great Britain as the country code identifier (included in the Western European GBD region) and the 2017 update of the GBD study as the base for recalibration parameters.

**World Health Organization cardiovascular disease (CVD) risk scores calculated using Health Survey for England data.**

**Laboratory based (1998, 2003, 2006, and 2009-2017)**

- Age (40-74 y)
- Sex
- Systolic blood pressure (SBP)
- Physician-diagnosed diabetes
- Current smoking
- Total cholesterol

**Non–laboratory-based (1998, 2001-2003, and 2005-2017)**

- Age (40-74 y)
- Sex
- SBP
- Current smoking
- BMI

###### Globorisk Score

The Globorisk algorithm calculates the predicted 10-year risk of CVD (CHD or stroke).

Risk prediction models were derived using individual participant data (aged ≥40 years with no baseline CVD, with a maximum follow-up of 15 years) pooled from 8 prospective United States–based cohorts. Cohort-specific models were developed for (1) fatal CVD and (2) fatal-plus-nonfatal CVD (for countries with available data on CVD incidence) using the same set of risk factors as described in the WHO Risk Score section. HRs were estimated using Cox proportional hazards models, including interaction terms to allow for age and sex differences in the effects of risk factors on CVD risk (eg, the estimated associations of diabetes and smoking were observed to be stronger in women) [36,37].

Using a similar process as described in the WHO Risk Score section, models were then recalibrated by applying the risk equation to national-level data on risk factor levels and CVD event rates to calculate the predicted 10-year CVD risk.

The laboratory-based Globorisk score calculated the predicted 10-year risk of CVD in adults aged 40 to 74 years using age, sex, SBP, diabetes (based on blood sugar levels or having a history of diabetes), smoking status, and total cholesterol [36,37]. The prediction was limited to those aged 40 to 74 years, as this age range is commonly used for assessment of primary prevention of CVD. The non–laboratory-based score replaces diabetes and total cholesterol with BMI. Globorisk scores are contemporarily recalibrated for the target country [36-38]; for our study, we specified the population of Great Britain and the baseline year of 2020 and calculated the risk scores for fatal-plus-nonfatal CVD. Globorisk scores for HSE participants were computed using the same analytical samples and risk factor definitions as for the WHO algorithms and were calculated using the R (version 4.2.2; R Foundation for Statistical Computing) package *Globorisk* [46].

##### CVD Risk Score Components

###### Age

All adults (defined as aged ≥16 years in the HSE series) selected in the general population sample in the relevant survey years, who completed the health interview, were included in the harmonized dataset. Since 2015, only categorical age (16-17 years, 18-19 years, and in 5-year intervals up to age ≥90 years) has been provided in the end-user license (EUL) datasets to preserve anonymity of participants. Continuous age (up to ≥90 years) was provided in the special license (SL) dataset for 2015 (SL data collections contain more detailed information than EUL data). For participants in the HSE 2016-2017, age in our study was set to the midpoint of categorical age (data under the 2016-2017 SL was not available at the time of writing this manuscript).

###### Cigarette Smoking Status

Participants were asked whether they had ever smoked a cigarette, and those who reported having ever smoked were asked whether they smoked cigarettes at all nowadays. Participants aged ≥25 years were asked about their smoking behavior during the interview. In the HSE series, participants are classified as current smokers, ex-smokers, or never smokers. A binary smoking variable (current smoker or not current smoker) was used in our study to calculate CVD risk.

###### Calculation of BMI

BMI data are derived from measured height and weight. Toward the end of the interview, height was measured by trained interviewers using a portable stadiometer with a sliding head plate, a base plate, and connecting rods marked with a measuring scale. Participants were asked to remove their shoes. One measurement (to the nearest even millimeter) was taken, with participants stretching to the maximum height and the head positioned in the Frankfort plane. For participants who were not pregnant, a single weight measurement (to the nearest 100 g) was recorded using digital scales. Participants were asked to remove their shoes and any bulky clothing or heavy items from their pockets. Individuals who were unable to stand or were unsteady on their feet were not measured. The participants who weighed >130 kg (>200 kg since 2011) were asked for their estimated weight due to concerns about the accuracy of the scales above these levels. (Class III Seca scales were introduced in the HSE 2011; these met a higher specification than previous [class IV] scales and measure up to a maximum of 200 kg.) Participants were assigned missing values if they were considered by the interviewer to have unreliable measurements, for example, those who were too stooped or wore excessive clothing. Height and weight measurements were voluntary. A sizeable and increasing number of participants had missing anthropometric data; our own analyses of HSE 2003-2018 data showed that the propensity to have missing values was associated with older age, lower educational status, and fair, bad, or very bad general health [47]. BMI was calculated as weight in kilograms divided by height in meters squared, and the WHO obesity classification was used to group participants into mutually exclusive categories [48].

###### SBP Measurement

BP was measured during the nurse visit using standardized protocols; Dinamap (Critikon) 8100 monitors were used before 2003, and Omron (Omron Healthcare Co Ltd) HEM 907 have been used since. Dinamap readings were converted into Omron readings using a regression equation based on a calibration study [49]. Three BP readings were taken from each participant while seated, at 1-minute intervals, using an appropriately sized cuff on the right arm, if possible, after a 5-minute rest. Measurements from participants who had exercised, eaten, drunk alcohol, or smoked in the 30 minutes before measurements were recorded as not valid. The mean of the second and third valid SBP readings was used in our study.

###### Treatment for High BP

Use of antihypertensive medication is a component of the Framingham Risk Scores [34]. Nurses recorded the details of any classes of medication for high BP that participants reported taking at the time of the survey. Since 2003, participants taking medicines that lower BP were asked whether they were taking the medicine because of a heart problem, high BP, or for some other reason. Two different definitions of use of BP medicine are therefore available [50]. First, participants can be classified as being on treatment if the BP medicine they were taking was prescribed specifically to treat their BP. Second, participants can be classified as being on treatment if they were taking any medicines commonly used to treat high BP, regardless of whether the medicines were reported by the participant as being prescribed for that reason. The former (more restrictive) definition has been used in the HSE series from 2003 onward to classify participants as having survey-defined hypertension (ie, SBP ≥140 mm Hg or diastolic BP ≥90 mm Hg or taking medicine prescribed for high BP) [51].

###### Diabetes

The item on physician-diagnosed diabetes was included in the main interview in 1998, 2003, 2006 (all adults aged 16-64 years, but a random half of those aged ≥65 years), and each year from 2009 onward. The interview made no distinction between type 1 and type 2 diabetes. In addition, HbA_1c_ levels were measured in nonfasting blood samples collected at the nurse visit. HbA_1c_ reflects average blood sugar levels over the previous 2 to 3 months and can therefore be used both to monitor diabetic control in people with diagnosed diabetes and to detect undiagnosed diabetes [52]. In the HSE series, HbA_1c_ values expressed as a percentage were available in 2003, 2005 to 2006, and from 2008 onward; HbA_1c_ levels reported in SI units of mmol/mol were available from 2012 onward. The latter is currently used in the annual HSE Adult Health reports to define total diabetes, which is characterized by an HbA_1c_ level of ≥48 mmol/mol (diagnostic of diabetes) or self-reported diagnosed diabetes [53]. Due to changes in calibrators, HbA_1c_ values were adjusted upward from the fourth quarter of fieldwork for the HSE 2013 onward to ensure comparability with earlier years. In our analyses (not presented herein), HbA_1c_ values expressed as a percentage (nonoutlying values: between 2.5% and 24.9%) were converted to mmol/mol values using a conversion equation [54].

###### Total Cholesterol

Cholesterol levels were measured via nonfasting blood samples taken at the nurse visit. Due to a change in calibrators, cholesterol levels between 2011 and 2014 were adjusted downward to ensure comparability with values from earlier years. A further change in calibrators in 2015 resulted in equivalence between the measurements in current years and those before 2010.

##### Harmonized Variables to Adjust for Change in Measuring Equipment

To avoid duplication of effort, we have provided variables in the harmonized dataset that researchers can use to suitably adjust for the changes over time in the machinery used in the HSE to measure BP, total cholesterol, and HbA_1c_. These are shown in Table 1.

**Table 1:** Harmonized variables to adjust for changes in measuring equipment.

CVD^a^ risk factor			Adjustments		Harmonized variable
**BP^b,c^**					
	Systolic BP	8.90 + (Dinamap × 0.91)		omsysval	
	Diastolic BP	19.78 + (Dinamap × 0.73)		omdiaval	
**Total cholesterol^d^**			Unadjusted minus 0.1 mmol/L		cholval13
**HbA_1c_^e^(mmol/mol)^f^**					
	Lower range	16-41: +1 mmol/mol		glyhb2_h	
	Middle range	42-68: +2 mmol/mol		glyhb2_h	
	Higher range	≥69: +3 mmol/mol		glyhb2_h	

^a^CVD: cardiovascular disease.

^b^BP: blood pressure.

^c^Blood pressure was measured using standardized protocols with the use of Dinamap (Critikon) 8100 monitors before 2003 and Omron (Omron Healthcare Co Ltd) HEM 907 from 2003 onward. In the creation of the harmonized dataset, the pre-2003 Dinamap values were converted to Omron values using previously published regression equations based on a calibration study that derived predicted Omron readings from the observed Dinamap readings [49].

^d^New analytical equipment was introduced in April 2010 and June 2015 by the laboratory that carried out the analyses on the blood samples taken during the nurse visit, which resulted in a slight change in the reference range for total cholesterol. For the harmonized dataset, the laboratory values were adjusted downward by 0.1 mmol/L to be comparable to the values before April 2010. For the new equipment introduced post 2015, the laboratory values were on average 0.1 mmol/L lower than the equipment used between 2010 and 2015; hence, no adjustment was needed to be comparable to the values before April 2010 [55].

^e^HbA_1c_: glycated hemoglobin.

^f^A new calibration lot for the processing of glycated hemoglobin was introduced in September 2013. Comparisons by the manufacturer indicated that the new machinery produced lower values, necessitating upward adjustment to be comparable with values before the change in equipment [55].

##### Explanatory Variables for Changes in CVD Risk Over Time

###### Socioeconomic Status

Measures of individual-level socioeconomic status (SES) included educational status, social class, and household income. Educational status was classified into 4 categories according to the highest educational qualification: (1) university degree or equivalent, (2) A level or diploma, (3) O level, General Certificate of Secondary Education, or vocational equivalent, and (4) none. The occupational (social) class was determined using the registrar-general’s classification (professional, managerial technical, skilled nonmanual, skilled manual, semiskilled manual, unskilled manual, unemployed, and other or not fully described). The household reference person reported annual gross household income from all sources via a showcard with 31 income categories. Household income was equivalized by considering the number of adults and dependent children in the household (McClements scale [56]); households were divided into quintiles. Tenure, availability of a car, and number of cars normally available for use by household members are also included as other measures of individual-level SES.

Area-level SES was classified in the HSE datasets (from 2001 onward) according to the index of multiple deprivation (IMD). This is a composite index of relative deprivation at lower-layer super output area (LSOA) level, based on 7 domains of deprivation: (1) income, (2) employment, (3) health deprivation and disability, (4) education, skills, and training, (5) barriers to housing and services, (6) crime and disorder, and (7) living environment. LSOAs comprise between 400 and 1200 households and typically contain a resident population between 1000 and 3000 persons. LSOA boundaries remain fixed over time, ensuring that values of the IMD are comparable over time. National quintiles of area deprivation are created through ranking LSOAs according to their deprivation score. The postcode address of responding households in each survey was linked to the LSOA, which was then used to determine the corresponding deprivation quintile. The IMD was first included in the HSE 2004 dataset and was updated in 2007, 2010, and 2015; the HSE datasets available at the UK Data Service (and the harmonized dataset compiled for our study) contain the version of the IMD that was current at the time of each survey.

###### Behavioral Risk Factors: PA and Alcohol

In the HSE series, questions on PA assessed frequency (number of days spent doing a specified activity in the last 4 weeks) and duration (of an average episode lasting above a specified bout duration limit) in 4 leisure-time domains: domestic activity, do-it-yourself or manual work, walking, and sports or exercise. In the reporting of trends, PA undertaken while at work is also considered in the estimation of summary activity levels for HSE reports. PAs are classified into intensity levels (light, moderate, and vigorous) based on an estimate of the energy expenditure associated with each activity.

Changes in the PA questions (reflecting changes over time in policy recommendations, namely, the reference period for bouts of activities to report) have restricted the meaningfulness of comparisons over time to some extent. The lower duration limit for an activity to be included was 15 minutes in 1998 and 2006; 30 minutes in 2003 (15 minutes for sports and exercise); and 10 minutes in 2008, 2012, and 2016. A single question on occupational PA (“Thinking about your job, in general would you say that you are very physically active, fairly physically active, not very physically active, or not at all physically active?”) was asked in 2003 and 2006; more detailed questions introduced in 2008 (repeated in 2012 and 2016) focused on what people actually do at work (eg, climbing stairs or ladders, lifting, and carrying or moving heavy loads) and how many hours they typically work.

To maximize the trend series, we derived a variable summarizing the number of days per week that participants undertook PA of at least moderate intensity for a minimum duration of 30 minutes. For those participants who reported that they were very or fairly active in their job, arbitrary estimates of 12 or 20 working days in the last 4 weeks (3 or 5 days per week, respectively) were used, depending on whether the participant worked part time or full time, to assess levels of PA while at work.

The main interview included questions on the number of drinking days in the last week (collected in all years), alcohol consumption (type and quantity) on the heaviest drinking day in the last week (all years), and average weekly drinking over the past 12 months (2011 onward). Information on the type and quantity of drinks consumed were used to estimate alcohol unit consumption using a method of conversion detailed elsewhere [57]. The applied conversion factors were revised in 2006 to 2007 to account for changes to the drinking environment. Alcohol units were categorized to represent consumption on the heaviest drinking day relative to recommended daily limits at the time of the survey (>3 units for women and >4 units for men); binge drinking was defined as drinking twice the recommended daily limits (>6 units for women and >8 units for men) [58]. Additional variables classified participants according to whether they drink alcohol nowadays (2 categories: nondrinker and current drinker; 3 categories: never, former, and current drinker).

##### General Health and Long-Standing Illness

Participants were asked to rate their health in general (response options: very good, good, fair, bad, and very bad). Long-standing illnesses were also reported in the survey. Before 2012, the question on long-standing illness referred to “an illness, disability or infirmity...that has troubled you over a period of time or that is likely to affect you over a period of time.” Since 2012, long-standing illness is defined as “any physical or mental health condition or illness lasting or expected to last 12 months or more.”

##### Diagnosed CVD Conditions

The HSE surveys for 1998, 2003, 2006, 2011, and 2017 had a specific focus on CVD. During the interview, adults were asked a series of questions about whether they had ever been diagnosed with certain specified CVDs, and if so, whether the diagnosis had been made by a physician. The specified conditions included angina, myocardial infarction, stroke, abnormal heart rhythm, a heart murmur or “other cardiovascular condition.” No attempt was made to verify these self-reported diagnoses. Therefore, it is possible that some misclassification may have occurred because some participants may not have remembered, or may have misremembered, the diagnosis made by their physician.

##### Use of Medicines

At the nurse visit, participants were asked the following: “Are you taking or using any medicines, pills, syrups, ointments, puffers or injections prescribed for you by a doctor or nurse?” Those who did were then asked the name of each prescribed item. In most cases, participants showed the nurse the actual medicine pack. These were coded by the nurse into medicine classes based on the subsections of the British National Formulary. Up to 22 medicines could be recorded (this has recently increased to 32). For each medicine, a follow-up question asked whether they had taken or used that medicine in the last 7 days. Variables on the use of CVD medicines, lipid-lowering medicines, and BP-lowering medicines are provided in the harmonized dataset.

##### Pregnancy Status

At the nurse visit, women aged 16 to 49 years were asked whether they were pregnant at the moment.

##### Contraceptive Use

Some questions were completed by the participants in paper self-completion questionnaires. In the HSE 1998, 2001 to 2003, and 2005 to 2006, this included questions for women on whether they had ever taken the contraceptive pill or had a contraceptive injection or implant. Those replying yes were asked whether they were currently taking the contraceptive pill or having a contraceptive injection or implant. On the basis of these 2 questions, we created a three-category variable distinguishing between women who reported that they (1) had never taken the contraceptive pill or had a contraceptive injection or implant, (2) had ever taken but were not currently taking the contraceptive pill or having a contraceptive injection or implant, and (3) those currently taking the contraceptive pill or having a contraceptive injection or implant. In addition, the current use of oral contraceptives was recorded each year at the nurse visit in the use of medicines section.

##### Other Variables

Other sociodemographic variables compiled in the harmonized dataset included marital status (single, married, separated, divorced, widowed, and cohabitees), ethnic group (White, Black, Asian, mixed, and other), government office region (GOR: North East, North West, Yorkshire and the Humber, East Midlands, West Midlands, East of England, London, South East, and South West), an urban or rural indicator, and receipt of various means-tested state benefits (eg, Income Support and Housing Benefit).

##### Sampling Design Information (Primary Sampling Units, Strata, and Weights)

Using the small-user Postcode Address File as the sampling frame, a 2-stage stratified random sampling process was used to select each year’s general population sample. First, a random sample of primary sampling units (PSUs), based on postcode sectors, was selected, with probability proportional to the total number of addresses. Stratification was performed by ordering the PSUs according to local authority, and within each local authority by the percentage of households in the last census where the head of household was in a nonmanual occupation. The list of PSUs was then sampled at fixed intervals from a random starting point. Second, a random sample of a fixed number of addresses was then drawn from each PSU, ensuring a self-weighted design in which every eligible participant had the same probability of selection.

Each pair of PSUs in the ordered list was assigned to the same stratum. Since 2006, the Taylor series method (linearization) has been used in annual HSE reporting for variance estimation using the PSU and stratum identifiers. For the analyses of data pooled over several years, GOR has often been used as an alternative stratification variable.

In 2003, weighting the general population adult sample for nonresponse was introduced for the first time in the HSE series [59]. The nonresponse weights take account of nonresponse at 4 levels: household response, individual response to the interview, individual response to the nurse visit, and individual response to the collection of blood samples. The harmonized dataset includes the relevant interview, nurse, and blood sample weights for each survey year from 2003 onward. These weights are scaled so that their sum over the relevant set of participants equals the unweighted sample size (resulting in an average weight of 1); the weighting variables before 2003 were assigned the value 1.

## Results

### Analytical Samples

**Figure 1:**
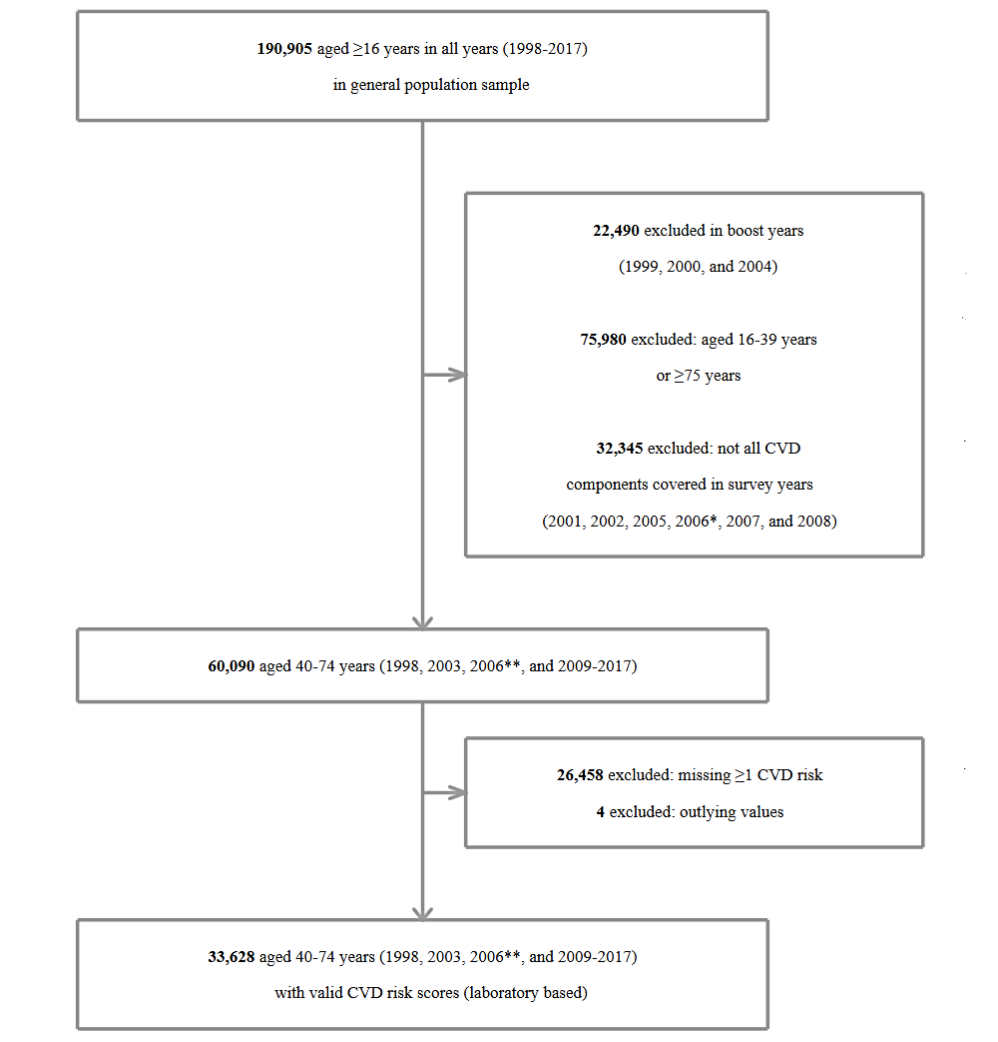
Flowchart of participants included in the estimation of changes over time in cardiovascular disease (CVD) risk (laboratory-based scores).*Allocated to physical activity module; **allocated to CVD (including diabetes) module.

**Figure 2:**
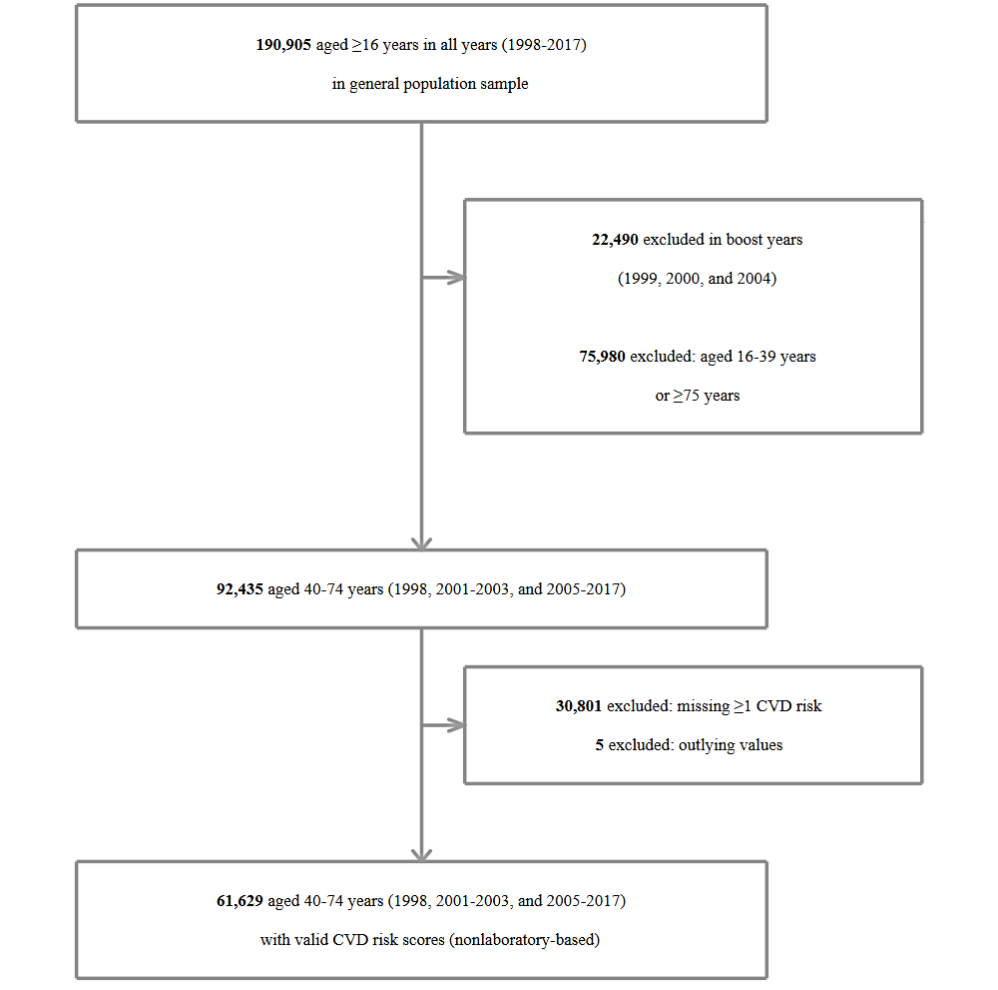
Flowchart of participants included in the estimation of changes over time in cardiovascular disease (CVD) risk (non–laboratory-based scores).

A total of 190,905 adults (aged ≥16 years) from the general population samples completed the health interview between 1998 and 2017 (Figures 1 and 2). The harmonized dataset excludes the participants in the boost years of HSE 1999, 2000, and 2004 (22,490/190,905, 11.78%) but includes the boost sample of adults aged ≥65 years in HSE 2005 (2673/193,578, 1.38%), resulting in a provided dataset of 88.38% (171,088/193,578) adults. Excluding the boost sample of adults aged ≥65 years in HSE 2005 for this study produced a dataset of 168,415 (nonboost sample) adults, of which 75,980 (45.12%) were excluded from the analyses due to falling outside the age range of 40 to 74 years.

### Missing Data on CVD Risk Scores

As shown in Figures 1 and 2, in the years when all CVD risk components were included in the survey, a sizeable number of adults aged 40 to 74 years were excluded from the analyses due to missing data on at least 1 risk component (30,801/92,435, 33.32% and 26,458/60,090, 44.03% for the non–laboratory-based and laboratory-based risk scores, respectively). The calculation of CVD risk scores requires complete (ie, nonmissing) risk factor information. As SBP is a component of both algorithms, inclusion in the analytical samples for calculating CVD risk is contingent on participants having participated in the nurse-visit stage of the survey and having their BP measured. In addition, as total cholesterol is a component of the laboratory-based scores, inclusion in this analytical sample is contingent on participants providing a nonfasting blood sample. Nonparticipation in the nurse visit and blood sample collection is therefore the main driver for the amount of missing data shown in the final stage of the flowcharts provided in Figures 1 and 2. An additional factor contributing to missing data for the non–laboratory-based scores is missing BMI data, due to refusals to undergo weight measurement during the health interview.

For the participants with complete and valid (ie, nonoutlying) data on each individual risk component, laboratory-based and non–laboratory-based 10-year CVD risk scores were calculated (33,628/60,090, 55.96% and 61,629/92,435, 66.67% participants aged 40 to 74 years, respectively). On the basis of unweighted data, the mean age of participants with laboratory-based scores was 56.1 (SD 9.8) years; 54.11% (18,197/33,628) of the participants were female. The sociodemographic profile was similar for those with non–laboratory-based scores.

### Analysis Plan

Analyses were performed separately by sex, given notable differences in CVD risk. These were conducted using Stata (version 18.0; StataCorp) with survey analysis procedures to account for the complex survey design (PSUs; GOR [strata]; and appropriate nonresponse weights, ie, nurse weights for the non–laboratory-based sample and blood sample weights for the laboratory-based sample).

**Figure 3:**
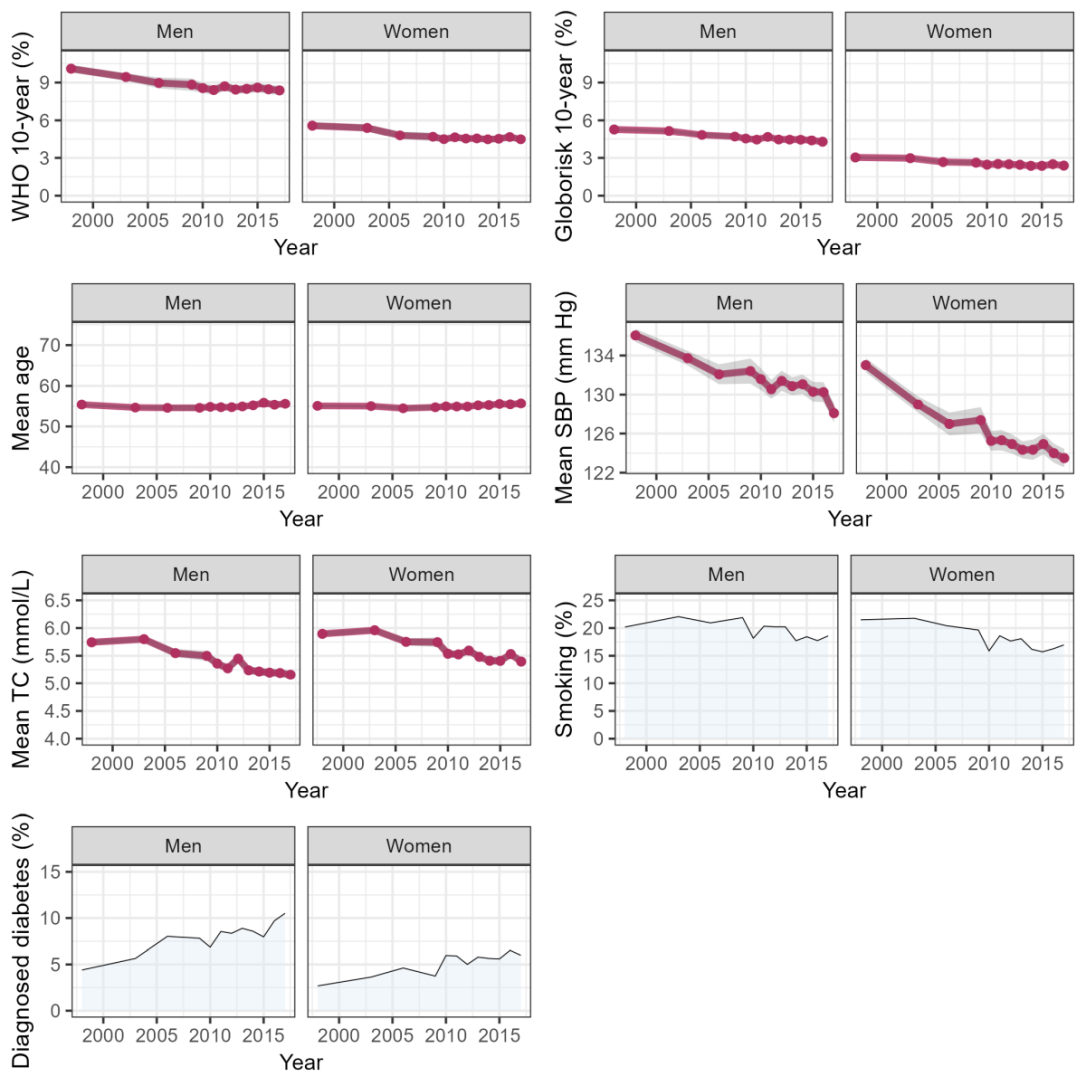
A 10-year cardiovascular disease (CVD) risk score (laboratory based) and its components by survey year and sex. SBP: systolic blood pressure; TC: total cholesterol; WHO: World Health Organization.

**Figure 4:**
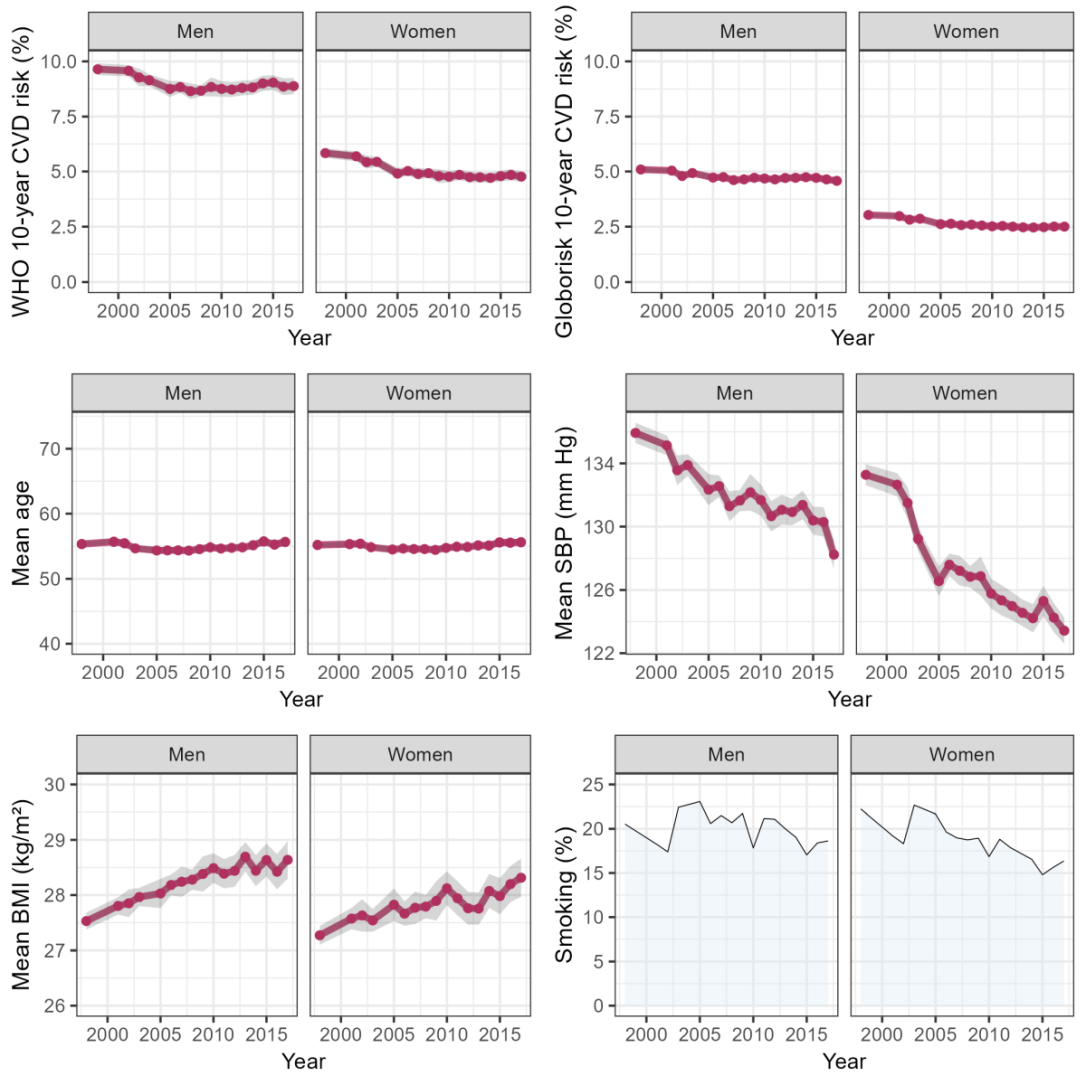
A 10-year cardiovascular disease (CVD) risk score (non–laboratory-based) and its components by survey year and sex. SBP: systolic blood pressure; WHO: World Health Organization.

For each survey year, we estimated the percentages (diagnosed diabetes and current smoking) and means of the individual risk components and the mean predicted 10-year risk of CVD (Figures 3 and 4). Wald tests were performed to test the null hypothesis of no change in the mean predicted 10-year risk of CVD between the first and last survey periods (1998 and 2017, respectively). Linear trends in CVD risk were tested using linear regression, with the predicted risk score as the outcome and survey year (continuous variable) as the independent variable. Statistical tests were 2-sided, and *P*<.05 was considered statistically significant.

### Trends in CVD Risk

The mean predicted 10-year CVD risk declined significantly over the last 2 decades in both sexes (for Wald tests, all *P*≤.001; for linear trend, all *P*<.001; Table 2). In men, the mean of the laboratory-based WHO risk score was 10.1% (SE 0.2%) and 8.4% (SE 0.2%) in 1998 and 2017, respectively; corresponding figures in women were 5.6% (SE 0.1%) and 4.5% (SE 0.1%). In men, the mean of the non–laboratory-based WHO risk score was 9.6% (SE 0.1%) and 8.9% (SE 0.2%) in 1998 and 2017, respectively; corresponding figures in women were 5.8% (SE 0.1%) and 4.8% (SE 0.1%). Globorisk risk scores were lower in absolute terms, but the pattern of change was very similar (for linear trend, all *P*<.001).

**Table 2:** Estimated linear trend in 10-year cardiovascular disease risk, Health Survey for England data (1998-2017).

			WHO^a^				Globorisk		
			β (%^b^; 95% CI)		*P* value^c^		β (%; 95% CI)		*P* value
**Laboratory based**									
	Men	–0.09 (–0.11 to –0.07)		<.001		–0.05 (–0.06 to –0.05)		<.001	
	Women	–0.06 (–0.08 to –0.05)		<.001		–0.04 (–0.04 to –0.03)		<.001	
**Non–laboratory based**									
	Men	–0.04 (–0.06 to –0.03)		<.001		–0.02 (–0.03 to –0.02)		<.001	
	Women	–0.06 (–0.07 to –0.05)		<.001		–0.03 (–0.04 to –0.03)		<.001	

^a^WHO: World Health Organization.

^b^Linear trends in CVD risk were tested using linear regression (accounting for the complex survey design), with the risk score as the outcome and survey year (continuous variable) as the predictor. The slope (β coefficient) represents the estimated annual decrease in the mean 10-year CVD risk (in absolute terms, expressed as a percentage). For example, for the laboratory-based WHO algorithm, the estimated annual decrease in the predicted 10-year CVD risk for men was 0.09% (eg, from 9.94% in 1998 to 9.85% in 1999).

^c^*P* value for linear trend.

### Trends in CVD Risk Components

The significantly declining linear trends in the mean predicted 10-year CVD risk reflected the net effect of diverging trends in its risk components. On the one hand, the data showed significant declines between the first and last survey periods in mean SBP (2017 vs 1998: declines of 8 mm Hg and 10 mm Hg in men and women, respectively), mean total cholesterol (0.6 mmol/L and 0.5 mmol/L), and lower levels of current smoking (decrease of 5 percentage points [PPs] in women; for Wald tests, all *P*≤.001; except *P*=.002 for smoking in women). Simultaneously, significant increases occurred in mean BMI (2017 vs 1998: increases of 1.1 kg/m^2^ and 1.0 kg/m^2^ in men and women, respectively) and levels of diagnosed diabetes (6 PPs and 3 PPs in men and women, respectively; for Wald tests, all *P*≤.001).

## Discussion

### Principal Findings

As CVDs remain the leading cause of death globally, using nationally representative health surveys from a high-income country such as England to model temporal trends in CVD risk can provide guidance for middle-income countries such as South Africa to inform where best to intervene and direct resources to reduce disease burden.

Modeling temporal trends in CVD risk requires pooling annual cross-sectional health surveys. Compiling and appending data from repeated cross-sectional surveys to enable such modeling is a daunting task due to changes in aspects such as survey content, question wording, inclusion of boost samples, weighting, measuring equipment, and guidelines for data protection. While data harmonization across aging cohorts such as the US Health and Retirement Study and the English Longitudinal Study of Ageing has benefitted enormously from the efforts of the Gateway to Global Aging team (including the production of harmonized datasets) [60], no such platform exists to enable researchers to harmonize data across repeated cross-sections of health examination surveys such as the HSE. In this manuscript, we have documented the methods and procedures used to painstakingly compile the harmonized dataset based on 17 years of separate HSE datasets spanning 2 decades (1998-2017), including a description of how we calculated the predicted 10-year risk of CVD using the WHO [35] and Globorisk [36-38] CVD risk algorithms.

In our presentation of early results, we showed significant declines over time in the mean predicted 10-year total (ie, fatal and nonfatal) CVD risk in both sexes, suggesting an improvement in cardiovascular health at the population level, consistent with modeling studies in England pointing to the role of increased prevention and treatment [61,62]. The observed trends in CVD risk reflect the net effect of divergent trends in its risk components, namely, significant declines in average levels of SBP, total cholesterol, and current smoking (women only), with simultaneous increases in mean BMI and diagnosed diabetes. This complex pattern of temporal trends in the individual CVD risk components agrees with other studies using HSE data over the same period [63].

### Implications of Our Findings

In the later stages of the EXPOSE study, more complex regression techniques will be used to compare trends in CVD risk between South Africa and England and empirically test the relative contributions of a wide set of factors that may explain those trends, including demographic, behavioral, social, environmental, and health care–related aspects. How the findings of this study apply to different countries is likely to be influenced by socioeconomic structures and health care systems (eg, access to health care is free at the point of use in the United Kingdom). Bearing this caveat in mind, our initial findings on the significant declines in 10-year CVD risk over 2 decades, accompanied by the conflicting trends in its modifiable risk components, can be leveraged to inform public health policy and interventions in the United Kingdom and in low- and middle-income countries such as South Africa with high CVD burdens.

First, our descriptive analyses show that the significant declines in the predicted 10-year risk for CVD may be attributable in large measure to population-level declines in cigarette smoking and in mean levels of BP and total cholesterol. In the absence of increasing levels of diagnosed diabetes and BMI, predicted risk would have declined at a stronger pace.

Second, the favorable trends in CVD risk demonstrates the population-level gains in cardiovascular health that are achievable through implementing a wide range of population-based public health primary and secondary prevention approaches. These include (1) policy and regulatory measures (eg, tobacco taxation and antismoking legislation, including smoke-free workplaces and public places); (2) public health campaigns promoting awareness about lifestyle behaviors (eg, diet and exercise); and (3) improvements in the early detection and management of CVD-related conditions such as hypertension, dyslipidemia, and diabetes through initiatives such as the National Health Service Health Check program and financial incentivization of general practices in screening for individual CVD risk factors (eg, increasing use of antihypertensive medicines and statins). Building on these successes, low- and middle-income countries could adopt similar approaches, adjusting for local socioeconomic and cultural contexts.

Third, evidence on the increasing levels of diagnosed diabetes and BMI shows that substantial challenges remain in reducing the CVD burden, and this can be used to leverage the expansion of prevention efforts to include combined lifestyle interventions to improve diets, levels of PA, and achieve sustained weight loss.

Finally, our study demonstrates the availability of long-standing, high-quality, nationally representative health examination survey data in high-income countries such as England to monitor population trends in CVD risk and its components, offering valuable evidence to inform public health policy, guide resource allocation, design targeted prevention strategies, and assess their effectiveness. Building similar capacity in population health surveillance in low- and middle-income countries is a major challenge due to factors such as budgetary constraints [64], but such investment would greatly contribute to identifying priorities for CVD prevention and evaluating the success of interventions.

### Strengths and Limitations

Our study uses high-quality data on the individual components of CVD risk, including objective measurements of BP, total cholesterol, and BMI, which avoids the potential inaccuracies of self-reported measures. Participants from health examination surveys such as the HSE are not selected on the basis of health care use, thereby increasing representativeness and avoiding selection bias to some extent. The harmonized dataset covers a time span of 2 decades, enabling modeling of temporal trends in CVD risk and investigation of which factors explain the trends. Area-level variables such as relative deprivation and urbanicity are also provided with the dataset, permitting analysis of contextual effects.

Although the authors of this study have considerable experience in collecting and analyzing HSE data, creating a harmonized dataset was a daunting task. The accuracy of variable derivation (eg, appropriate recoding to ensure congruence of the values across datasets) was checked by comparing estimates with the available trend tables published in annual HSE reports. We hope that the dissemination of our methods and procedures as well as the provision of code for harmonizing and appending the annual datasets will support future efforts by the wider research community.

Limitations of our study include increasing levels of nonresponse and reliance on complete case analyses in our presentation of early results (possibly biasing results). As mentioned earlier, the calculation of CVD risk scores requires complete (ie, nonmissing) risk factor information, and this approach is consistent with the model derivation stage of algorithms such as the WHO and Globorisk, which excluded participants with missing data on any of the selected risk factors.

As age in single-year intervals is no longer provided on the EUL datasets (to preserve the anonymity of participants), the calculation of predicted CVD risk using the midpoint of categorical age (in 5-year intervals) for participants in HSE 2016 to 2017 has inevitably reduced precision to some extent. A final limitation of our study is the cross-sectional nature of the HSE design, which prevents any validation of the risk algorithms (in the absence of appropriate data linkages).

### Conclusions

Monitoring temporal trends in predicted CVD risk and its risk factors at the population level is vital to support prevention efforts. Alongside evidence from longitudinal databases, harmonized data from repeated cross-sectional nationally representative health surveys can be used to identify and quantify the drivers of recent changes in CVD risk.

## Abbreviations

BP: blood pressure
CHD: coronary heart disease
CVD: cardiovascular disease
EUL: end-user license
EXPOSE: Explaining Population Trends in Cardiovascular Risk: A Comparative Analysis of Health Transitions in South Africa and England
GBD: Global Burden of Disease
GOR: government office region
HbA1c: glycated hemoglobin
HR: hazard ratio
HSE: Health Survey for England
IMD: index of multiple deprivation
LSOA: lower-layer super output area
PA: physical activity
PP: percentage point
PSU: primary sampling unit
SBP: systolic blood pressure
SES: socioeconomic status
SL: special license
WHO: World Health Organization
